# Application of imputation methods to the analysis of rheumatoid arthritis data in genome-wide association studies

**DOI:** 10.1186/1753-6561-3-s7-s24

**Published:** 2009-12-15

**Authors:** Douglas K Childers, Guolian Kang, Nianjun Liu, Guimin Gao, Kui Zhang

**Affiliations:** 1 Section on Statistical Genetics, Department of Biostatistics, University of Alabama at Birmingham, Birmingham, Alabama 35294, USA

## Abstract

Most genetic association studies only genotype a small proportion of cataloged single-nucleotide polymorphisms (SNPs) in regions of interest. With the catalogs of high-density SNP data available (e.g., HapMap) to researchers today, it has become possible to impute genotypes at untyped SNPs. This in turn allows us to test those untyped SNPs, the motivation being to increase power in association studies. Several imputation methods and corresponding software packages have been developed for this purpose. The objective of our study is to apply three widely used imputation methods and corresponding software packages to a data from a genome-wide association study of rheumatoid arthritis from the North American Rheumatoid Arthritis Consortium in Genetic Analysis Workshop 16, to compare the performances of the three methods, to evaluate their strengths and weaknesses, and to identify additional susceptibility loci underlying rheumatoid arthritis. The software packages used in this paper included a program for Bayesian imputation-based association mapping (BIMBAM), a program for imputing unobserved genotypes in case-control association studies (IMPUTE), and a program for testing untyped alleles (TUNA). We found some untyped SNP that showed significant association with rheumatoid arthritis. Among them, a few of these were not located near any typed SNP that was found to be significant and thus may be worth further investigation.

## Background

Advances in the understanding of a disease's pathogenesis often lead to improvements in strategy for the prevention, diagnosis, and/or treatment of the disease. Moreover, studies have shown that genetic factors play an important role in the pathogenesis of many complex human diseases. Therefore, improving public health and preventing disease provides sufficient motivation for dissecting the genetic etiology of complex human diseases. The genome-wide association study (GWAS) may be seen as a first step towards such dissections and have drawn considerable attention (with some success) in recent years.

Indeed, many GWAS have resulted in identifying at least one candidate gene that may seem likely, considering the biological properties of the gene, to have an effect on the disease [1]. In a typical GWAS, a large number of population samples of cases and controls are genotype at hundreds of thousands of single-nucleotide polymorphisms (SNPs). However, even at these numbers, the SNPs that are genotyped in GWAS will only account for a small proportion of cataloged SNPs. In particular, it is likely that disease susceptibility variants are not directly assayed. With the availability of a high-density panel of SNPs such as from HapMap [2], it is possible to gain additional power by testing untyped SNPs based on data at the genotyped SNPs. Testing untyped SNPs can facilitate the selection of SNPs to be genotyped in follow-up studies and can allow for comparison of findings or joint analysis of data from different studies that use different SNP panels and genotyping platforms.

Several methods have recently been developed and their corresponding software packages implemented to test untyped SNPs [3-5]. Although these methods differ in specific strategies used to impute genotypes at untyped SNPs, they generally follow three steps. In the first step, linkage disequilibrium (LD) patterns are dissected and/or haplotypes and their frequencies are inferred from genotypes of reference samples, such as genotypes from the HapMap project. In the second step, genotypes at untyped SNPs are imputed based on genotypes in observed data and their correlation with typed SNPs in reference samples. In the final step, association tests are performed on all typed and untyped SNPs. In this paper, we selected three software packages based on imputation methods, including Bayesian imputation-based association mapping (BIMBAM), imputing unobserved genotypes in case-control association studies (IMPUTE), and testing untyped alleles (TUNA) to analyze data from a GWAS of rheumatoid arthritis (RA) from North American Rheumatoid Arthritis Consortium (NARAC) provided to Genetic Analysis Workshop 16 (GAW16). These software packages were selected in this study because they are publicly available and can readily perform imputations and association tests in a genome-wide scale. We report our findings, compare the performances of the three programs, and discuss their advantages and disadvantages.

## Methods

### Data Sets

The case-control data was obtained from the NARAC provided for GAW16. It contains genotypes of NARAC (868 cases and 1,194 controls at 545,080 SNPs) after removing duplicated and contaminated samples. Because the three software packages were implemented for autosomes, only SNPs from 22 autosomes were used. SNPs with minor allele frequency (MAF) less than 0.01 and SNPs with *p*-value of Hardy-Weinberg equilibrium test in controls less than 0.0001 were removed. A total of 515,050 SNPs remained in our analysis. The Phase II genotype data of 60 CEU samples from the HapMap project http://www.hapmap.org/ was downloaded and used as reference data to impute genotypes at untyped SNPs.

### BIMBAM

BIMBAM [6] uses the methods implemented in fastPHASE [5] to impute the genotypes at untyped SNPs. The Bayes factors (BFs) are computed under linear or logistic regression of phenotypes on genotypes. Specifically, for binary (0/1) phenotypes, the BFs are computed under a logistic regression model, logit(Pr(*Y*_*i *_= 1)) = log(Pr(*Y*_*i *_= 1)/Pr(*Y*_*i *_= 0)) = *μ *+ *aX*_*i *_+ *dI*(*X*_*i *_= 1), where *Y*_*i *_denotes the phenotype for individual *i*, *X*_*i *_denotes the genotype for individual *i *(coded as 0, 1, or 2), *a *denotes the additive effect, and *d *denotes the dominance effect. The BFs are computed under the same priors for *μ*, *a*, and *d *as in prior D_2 _[5].

### IMPUTE

The computer program IMPUTE [7] uses a hidden Markov Model to determine the genotype probabilities for each individual in the study at untyped SNPs that are available in reference samples. The Cochran-Armitage trend test for associations is then implemented on the resulting file using the software SNPTEST [3]. A key feature of IMPUTE is that it can use genotype probabilities rather than deterministic genotypes. The test of additive association was used in our analysis.

### TUNA

The imputation-based analysis implemented in TUNA [8] uses a multi-locus measure of LD, similar in interpretation to *r*^2^, to determine the best set of genotyped markers that can be used for estimating the genotype frequencies of an untyped SNP in cases and controls separately. The statistical test for association implemented in TUNA aims to find differences in the allele frequencies in cases and controls [4]. It has an asymptotic chi-square distribution with one degree of freedom under the null hypothesis.

### Simulation study

Simulations were performed based on haplotypes and their frequencies of CHI3L2 gene from CEU and YRI samples from the HapMap. After removing SNPs with MAF less than 0.05, 25 and 17 SNPs were remained for CEU and YRI samples, respectively. One SNP with MAF of about 0.15 was selected as disease SNP and 400 cases and 400 controls were generated based on the genotypic relative risk of 1.0 (to assess the type I error) or 1.5 (to assess power) and a prevalence of 0.10. Genotypes at disease SNP and half of SNPs were removed to test the performance of imputation methods. The Phase II data of CEU 60 samples was used as reference, which allows us to investigate the performance of imputation methods when the LD pattern was misspecified. The simulation procedure was repeated 1,000 times.

## Results

The final number of typed and untyped SNPs we tested and the number of significant SNPs are listed in Table 1. Note that all three software packages automatically removed some SNPs from the analysis; the total number of typed SNPs tested was less than the total number of typed SNPs remaining after the preprocessing step. For IMPUTE and TUNA, the Bonferroni correction was used to determine significant SNPs. Specifically, a SNP was significant if its *p*-value was less than 0.05/*m*, where m was the number of SNPs tested, which was the total number of typed and imputed untyped SNPs and was different for different methods. We also set *m *as the number of typed SNPs tested to see how many typed SNPs were significant if only typed SNPs were included in the analysis. For BIMBAM, a SNP was significant if (BF) was bigger 3.5, which was comparable to the log_10_(BF) of 3.2 used by Servin and Stephens [5]. Several findings emerge from Table 1. First, notice that TUNA only tested a small number of untyped SNPs. This is because TUNA only tests untyped SNPs having strong LD with the typed SNPs. Second, all three methods found a substantial number of significant untyped SNPs. Third, chromosome 6 contains the major histocompatibility complex (MHC) region and a large number of significant SNPs were found on this chromosome. However, there were still a substantial number of significant typed and untyped SNPs even after excluding significant typed and untyped SNPs on chromosome 6. We also noticed that more significant SNPs were found with BIMBAM (201 with BIMBAM versus 43 with IMPUTE). One reason is that the use of log_10_(BF) of 3.5 to select significant SNPs with BIMBAM may not be stringent enough to correct the multiple testing problem in this situation. The appropriate threshold for BF needs to be further investigated. Fourth, some untyped SNPs were found to be significant from at least two methods and most of them were on chromosome 6. When considering the untyped SNPs on the 21 autosomes (not including chromosome 6), only 36 untyped SNPs were found to be significant by both BIMBAM and IMPUTE. The set of significant untyped SNPs identified by IMPUTE and TUNA did not overlap. Fifth, one may think the inclusion of untyped SNPs in the test will impose a serious multiple testing problem. It was true that the number of significant SNPs was reduced due to the conservative Bonferroni correction when using imputed SNPs compared to only using typed SNPs. However, this number was only reduced 17.6% for IMPUTE (from 428 to 364) and 4.2% for TUNA (from 371 to 356). Given that the follow-up study may only consider at most the top tens of SNPs, such reduction will not affect our selection in next step. Sixth, after excluding SNPs from chromosome 6 for typed SNPs, 44, 55, and 96 SNPs were found to be significant by BIMBAM, IMPUTE, and TUNA, respectively. Among them, 8 SNPs were found to be significant by both BIMBAM and IMPUTE, while 53 SNPs were found to be significant by both IMPUTE and TUNA. One reason for differences between BIMBAM and IMPUTE (or TUNA) is that BIMBAM uses a Bayesian approach that depends on priors while the tests in IMPUTE and TUNA are only slightly different from each other. We found 96 significant SNPs with TUNA with *p*-values similar to those calculated by IMPUTE. In addition, the *p*-values of about 40 SNPs from TUNA were marginally significant while their *p*-values were only marginally insignificant with IMPUTE. Such difference is due to the slightly different tests for association in TUNA and IMPUTE.

**Table 1:** Results from the analysis of rheumatoid arthritis data

	Number of SNPs			Number of Significant SNPs		
		
Methods	BIMBAM	IMPUTE	TUNA	BIMBAM	IMPUTE	TUNA
Results before significant SNPs on chromosome 6 removed						
Typed	499,102	515,049	499,084	469	364(428)^a^	411(431)^a^
Untyped	2,052,389	2,030,768	236,672	2,025	1,230	139
Total	2,551,491	2,545,817	735,756	2,494	1,594	550
Results after significant SNPs on chromosome 6 removed						
Typed	465,977	480,591	465,961	44	55(79)^a^	96(105)^a^
Untyped	1,902,577	1,882,164	217,913	201	43	16
Total	2,368,554	2,362,755	683,874	245	98	112

^a^For the number of significant SNPs identified in parentheses, m was set as the number of typed SNPs in the Bonferroni correction

Next, we consider an apparent relationship between the significant typed and significant untyped SNPs found by each method. In particular, for each method, the majority of untyped SNPs that were significant had nearby genotyped SNPs that also returned significant *p*-values for the same method. Especially for significant untyped SNPs on chromosome 6, all of them had nearby significant typed SNPs that were less than 100 kb apart. This observation indicates that using these methods may not sufficiently improve power to identify novel genes associated with RA. Although these SNPs may not be helpful for identifying novel genes that are associated with RA from the current study, they may be selected as candidate SNPs for follow up studies. Nevertheless, each method still found a few significant SNPs that were untyped and that are not located near a typed SNP that was also found to be significant. The significant untyped SNPs that are not located within 2 Mb of a significant typed SNP are listed in Table 2. Although these SNPs are not located on any previously identified genes that are associated with RA, they may be worth further investigation to identify novel genes associated with RA. For example, one SNP (rs9434795) located in gene *PLEKHG5 *was identified to be significant (*p*-value = 1.5 × 10^-9^) by IMPUTE; a previous study suggested that *PLEKHG5 *is associated with lower motor neuron disease [9]. Another two SNPs, rs4886414 and rs8042558, were found to be significant with BIMBAM and also had very small *p*-values with IMPUTE and TUNA.

**Table 2:** A list of significant untyped SNPs. The significant untyped SNPs that are not located with 2 Mb of any significant typed SNP.

				log_10 _(BF) or - log_10 _(*p*) (rank)^b^		
				
**Chr**.	RS Number	Distance^a ^(MB)	Gene	BIMBAM	IMPUTE	BIMBAM
15	rs4886414	41.38	SCAMP5	**4.07(84)^c^**	5.53(225)	6.35(24)
15	rs8042558	41.39	PPCDC	**4.06(85)**	5.53(226)	6.33(27)
2	rs10210993	37.45	MGAT5	**3.88(99)**	5.13(356)	Not Tested
2	rs10170556	37.41	MGAT5	**3.82(116)**	5.15(352)	Not Tested
2	rs2359965	35.03	KLF7	**3.57(183)**	6.17(102)	Not Tested
2	rs1263623	35.04	KLF7	**3.57(179)**	6.18(100)	Not Tested
2	rs1263624	35.03	KLF7	**3.57(184)**	5.13(358)	Not Tested
2	rs2244166	35.03	KLF7	**3.55(189)**	**5.78(176)**	Not Tested
2	rs2244399	35.03	KLF7	**3.54(195)**	**5.81(173)**	Not Tested
1	rs9434795	3.13	PLEKHG5	**0.94(20368)**	**9.94(6)**	Not Tested
21	rs2297248	2	USP25	0.13(162921)	0.02(1817334)	**9.04(7)**
11	rs7926700	14.32	DYNC2H1	-0.07(518517)	0.72(500481)	**7.23(14)**
20	rs6017239	4.99	TOX2	-0.08(575887)	0.05(1710287)	**9.56(3)**

^a^The distance to the closet typed SNP that was significant.

^b^Rank is among all untyped SNPs after excluding SNPs on chromosome 6.

^c^Bold font indicates significant SNPs.

The results from BIMBAM and IMPUTE based on simulation are presented in Table 3 because most of SNPs were somehow removed from the analysis with TUNA. For BIMBAM simulations, the thresholds of log_10_(BF) were set as log_10_(7.5) for the CEU samples and log_10_(6.5) for the YRI samples. Using these thresholds, BIMBAM had comparable type I error with IMPUTE using the Bonferroni correction. Several observations emerged from Table 3. First, the inclusion of untyped SNPs increased the power when the appropriate reference samples were used. The imputed MAF at untyped SNPs was close to the true value and BIMBAM had power comparable with IMPUTE. Second, the inclusion of untyped SNPs decreased the power when the inappropriate reference samples were used. Third, the type I error was still maintained for both BIMBAM and IMPUTE even when the inappropriate reference samples were used.

**Table 3:** Type I error, power, and difference between MAF from imputed and simulated genotypes

		Untyped+Typed SNPs		Typed SNPs	
			
Sample		BIMBAM	IMPUTE	BIMBAM	IMPUTE
CEU	Type I	0.021	0.015	0.029	0.031
	Power	0.71	0.708	0.537	0.541
	Difference	9.6 × 10^-4^	6.2 × 10^-4^	N/A	N/A
YRI	Type I	0.032	0.039	0.029	0.033
	Power	0.144	0.155	0.176	0.195
	Difference	2.43 × 10^-2^	2.20 × 10^-2^	N/A	N/A

## Discussion

In this paper, we applied three imputation methods, BIMBAM, IMPUTE, and TUNA to a GWAS of RA data. All of these methods identified some untyped SNPs that showed significant association with RA. A few of these are also not located near a significant typed SNP. This provides some reason for being selected in follow-up studies to identity novel genes that are associated with RA.

Many imputation-based methods have recently been proposed, thus it is necessary to compare their strengths and weaknesses. Indeed, each of these methods has certain advantages over the other. For one, TUNA is computational efficient because it only uses a small number of typed SNPs to estimate the genotype frequencies of the untyped SNPs. Many of the untyped SNPs were not tested by TUNA. This further increases its efficiency-TUNA took only about 12 hours to finish all computations. However, TUNA may miss significant SNPs. BIMBAM uses Bayesian methods, runs efficiently and allows for the input and output of zipped files rather than large text files. The use of BFs has some advantages over the standard *p*-value approach [5] but results are not easily comparable with measures of significance. IMPUTE has the advantage of allowing the user to decide the test performed and use imputed genotype probabilities. However, IMPUTE took more than 400 hours to complete our computations. Such a heavy computational demand stands as a roadblock for its general application in GWAS.

We evaluated their performances in terms of the accuracy of imputed genotypes and the power and type I error rate of the subsequent association testing using simulated data. Our results indicated that when an inappropriate reference sample is used, the power may decrease but the type I error rate is maintained. However, when an appropriate reference sample is used, the genotypes at untyped SNPs were accurately imputed and the power was increased. Thus, in this case, it may be desirable to use these imputation methods. On the other hand, we found some untyped SNPs were identified as significant with one method but not with any of other methods from the analysis of real data. Therefore, results of untyped SNPs from these imputation methods must be used with caution.

## List of abbreviations used

BF: Bayes factors; BIMBAM: Bayesian Imputation-Based Association Mapping; GAW16: Genetic Analysis Workshop 16; GWAS: Genome-wide association study; IMPUTE: Imputing unobserved genotypes in case-control association studies; LD: Linkage disequilibrium; MAF: Minor allele frequency; MHC: Major histocompatibility complex; NARAC: North American Rheumatoid Arthritis Consortium; RA: Rheumatoid arthritis; SNPs: Single-nucleotide polymorphisms; TUNA: Testing untyped alleles

## Competing interests

The authors declare that they have no competing interests.

## Authors' contributions

DKC acquired the data, performed the statistical analysis, and drafted the manuscript. GK performed the data pre-processing and participated in the statistical analysis. KZ conceived of the study. NL, GG, and KZ participated in the design of the study and helped to draft the manuscript. All authors read and approved the final manuscript.
